# Oxidative Stress in Wistar Rats Under Acute Restraint Stress and Its Modulation by Antioxidants and Nitric Oxide Modulators

**DOI:** 10.7759/cureus.43333

**Published:** 2023-08-11

**Authors:** Giridhari Pal, Hara Prasad Mishra, Tarun Kumar Suvvari, Anshul Tanwar, Tamoghna Ghosh, Pankaj Verma, Abhilasha Pal, Kuldeep Patial, Chinmaya Mahapatra, Nidhal A Amanullah, Sara A Shukoor, Sibin Kamal, Vishwajeet Rohil

**Affiliations:** 1 Pharmacology, Vallabhbhai Patel Chest Institute, University of Delhi, Delhi, IND; 2 Clinical Trial, All India Institute of Medical Sciences, New Delhi, Delhi, IND; 3 Pharmacology and Therapeutics, University College of Medical Sciences, University of Delhi, Delhi, IND; 4 Medicine and Surgery, Squad Medicine and Research (SMR), Visakhapatnam, IND; 5 General Medicine, Rangaraya Medical College, Kakinada, IND; 6 Medicine, All India Institute of Medical Sciences, New Delhi, Delhi, IND; 7 Zoology, Miranda House, University of Delhi, Delhi, IND; 8 Sleep Medicine Division, Vallabhbhai Patel Chest Institute, University of Delhi, Delhi, IND; 9 School of Pharmacy, The Neotia University, Sarisha, IND; 10 Psychiatry and Behavioral Sciences, Sree Ramakrishna Mission Hospital, Thiruvananthapuram, IND; 11 Psychiatry, Government Medical College Trivandrum, Trivandrum, IND; 12 Pain and Palliative Medicine, IQRAA International Hospital & Research Centre, Kandhla, IND; 13 Biochemistry, Vallabhbhai Patel Chest Institute, University of Delhi, Delhi, IND

**Keywords:** nitric oxide modulators, wister rats, in vivo study, antioxidants, restraint stress

## Abstract

Background: Several pathogenic conditions leading to morbidity, including cancer, aging, diabetes, reperfusion injury, cardiovascular disease, and neurological disorders, are known to be exacerbated by oxidative stress. Antioxidant therapy is effective in the treatment of such disorders and appears to be a potential therapeutic technique to reduce oxidative stress. The aim of our study is to investigate the antioxidant effects of L-ascorbic acid and nitric oxide (NO) modulators on rats suffering from oxidative stress induced by acute restraint stress (RSx1).

Methodology: In this in vivo study, Wistar rats were subjected to one hour of restraint stress on day 21 to induce oxidative stress. Superoxide dismutase (SOD), total antioxidant capacity (TAC), catalase, glutathione (GSH), and malondialdehyde (MDA) were used to assess the antioxidant effects. IBM Corp. Released 2013. IBM SPSS Statistics for Windows, Version 22.0. Armonk, NY: IBM Corp. was used for data analysis.

Results: Compared to vehicle groups, acute restraint stress (RSx1) dramatically increased MDA levels while decreasing GSH, SOD, total antioxidant capacity, and catalase. L-NAME, 7-NI, AG (50 mg/kg each), and L-ascorbic acid (200 mg/kg) reversed the changes in SOD, MDA, GSH, total antioxidant capacity, and catalase levels. The NO precursor L-arginine (1000 mg/kg) and NO synthase inhibitors followed the same trend.

Conclusion: Our study findings highlight the complex role of antioxidants and NO modulators in the pathogenesis of diseases, as evidenced by the reversal of oxidative stress indicators. Antioxidant therapy, with its potential to mitigate oxidative stress, emerges as a viable treatment option for a range of pathological conditions associated with oxidative stress.

## Introduction

Stress is a major contributor to a range of mental and non-psychiatric disorders. From a basic science and therapeutic perspective, several different fields of knowledge are involved in the study of stress, from a genetic perspective to extensive behavioral testing and clinical evaluation. In the biotic environment, oxidative stress is caused by two factors: an excess production of reactive oxygen species (ROS) and a lack of adequate enzymatic and non-enzymatic antioxidants [1]. In other words, oxygen-dependent metabolic processes lead to oxidative stress, which disturbs the balance between pro- and antioxidants in living organisms. Excessive ROS can interfere with the proper functioning of cellular lipids, DNA, or proteins. Oxidative stress has been linked to several manifestations, including aging [2]. Living organisms must maintain the delicate balance between advantageous and disadvantageous impacts of free radicals, which is done through processes known as "redox regulation". By monitoring the redox status in vivo, the "redox regulation" process safeguards living things from a variety of oxidative stimuli and preserves "redox homeostasis" [3]. 

When present in large quantities, ROS can significantly affect cellular architecture, nucleic acids, lipids, and the protein damage cascade [4]. It is well known that the hydroxyl radical interacts with and damages the pyrimidine and purine bases and the deoxyribose backbone of DNA molecules. The primary step in mutagenesis, aging, and cancer is the permanent alteration of the genetic material caused by these "oxidative damage" events [5]. It is generally known that metal-induced ROS generation damages DNA as well as other biological elements, such as polyunsaturated fatty acid residues of phospholipids, which are highly susceptible to oxidation [6]. The by-product of the peroxidation process is malondialdehyde, when peroxyl radicals are reorganized into endoperoxides (precursors of malondialdehyde) by a cyclization reaction (MDA). The primary aldehyde produced by lipid peroxidation, besides malondialdehyde, is 4-hydroxy-2-nonenal (HNE). MDA is mutagenic in both mammals and bacteria and causes cancer in rats. Thus, the most dangerous consequence of lipid peroxidation appears to be hydroxynonenal, although it is only marginally mutagenic [7]. The processes behind protein oxidation by reactive oxygen species have been studied. All protein amino acid residues, such as cysteine and methionine residues, are susceptible to oxidation of their side chains by ROS. When cysteine residues are oxidized, low-molecular-weight thiols, such as GSH and protein thiol groups (-SH), can combine to form reversible mixed disulfides (S-glutathionylation). The concentration of carbonyl groups, which can be formed in a variety of ways, is a reliable predictor of the level of protein oxidation induced by ROS [8]. 

A variety of defense mechanisms have been developed by organisms when exposed to free radicals from different sources [9]. There are four types of defenses against free radical-induced oxidative stress: repair defenses, preventive defenses, antioxidant defenses and physical defenses. Enzymatic antioxidant defenses include catalase, glutathione peroxidase, and superoxide dismutase. Examples of non-enzymatic antioxidants include alpha-tocopherol, ascorbic acid, carotenoids, glutathione, flavonoids, and other antioxidants. Both the intracellular levels and the actions of these antioxidants are in balance under normal circumstances. The survival and health of living organisms depend on this balance [10].

Glutathione, a very abundant redox buffer and thiol antioxidant, is the primary soluble antioxidant in the cytosol, nucleus, and mitochondria [11]. Critical sulfhydryl proteins required for DNA expression and repair are maintained in a stable redox state by GSH in the nucleus. The amount of oxidized glutathione that accumulates in cells is a good predictor of how exposed an organism is to oxidative damage, as is the GSH/GSSG ratio. There is a risk of oxidative damage to several enzymes when GSSG levels are too high. Glutathione plays a key role in protective function against oxidative stress. It helps in transporting amino acids across the plasma membrane, and it acts as a cofactor for various detoxifying enzymes that counteract oxidative stress. Glutathione directly scavenges harmful hydroxyl radicals and singlet oxygen, effectively neutralizing hydrogen peroxide and lipid peroxides through the catalytic action of glutathione peroxidase. Additionally, glutathione has the capability to restore the activity of essential antioxidants, such as vitamins C and E. It achieves this by directly or indirectly reducing the tocopherol radical of vitamin E and converting semi-dehydroascorbate to ascorbate [12]. Protection against oxidative stress is also significantly aided by vitamins C and E, lipoic acid, carotenoids, and other non-enzymatic antioxidants, such as catalase, SOD, and glutathione peroxidase [13].

The relationship between stress and an increase in oxidant production, oxidative damage, and cellular damage caused by free radicals and their reaction intermediates is well established [14]. The severity, duration, and type of stressor all influence the amount of oxidative damage that occurs [15]. One of the first signs of the stress response is the release of glucocorticoids. Glucocorticoids can induce neurotoxicity in two different ways: by altering energy metabolism or by increasing excitatory amino acids, such as glutamate, in the extracellular space. Neuronal excitotoxicity can be induced by glutamate [16,17]. This chain of events leads to the activation and release of TNF-alpha convertase (TACE) in the brains of rats subjected to restraint stress [18]. Studies have shown that oxidative and inflammatory stress indicators in the brain may change as a result of restraint stress, although pre-treatment with gabapentin may be protective against these changes [19]. Some theories suggest that neural cells, including stem cells, are more susceptible to oxidative stress damage when exposed to high levels of glucocorticoids during pregnancy. In vitro/ex vivo experimental models have been used to determine which intracellular pathways in developing neurons, including cerebellar granule cells and neural stem cells, are triggered by oxidative stress [20]. Severe stress has been linked to Parkinson's disease because it can increase tetrahydrobiopterin and dopamine and damage dopamine neurons in vivo through oxidative stress [21]. Long-term immobilization of rats resulted in oxidative stress, further leading to increased lipid peroxidation and decreased levels of SOD, GST, catalase, and glutathione. Compared to vitamins A and C, vitamin E was found to be more effective in reducing lipid peroxidation, increasing SOD, GST, and catalase activity, and increasing glutathione levels after stress. To scavenge free radicals generated in brain tissue and reduce oxidative stress, vitamin E can be administered as a dietary supplement [22]. Therefore, the aim of our current study was to evaluate how antioxidants and NO modulators affect oxidative stress in Wistar rats during acute restraint stress.

## Materials and methods

Chemicals/drugs and animals

**Figure 1:**
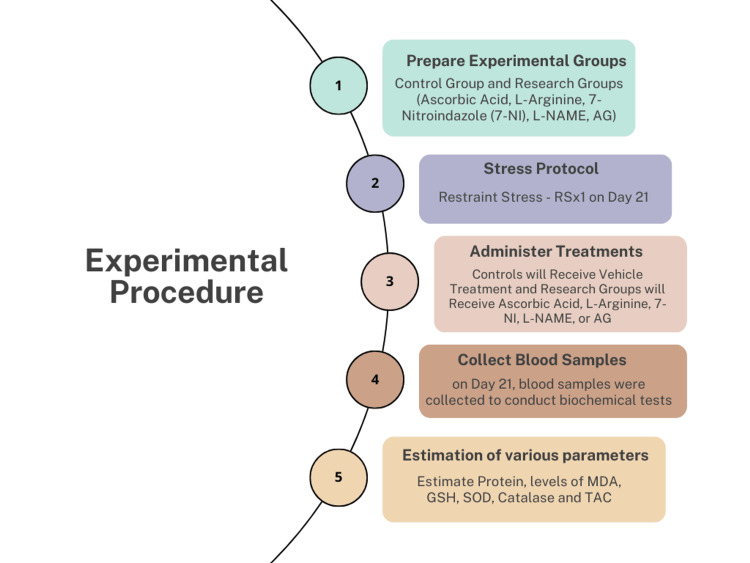
Experimental procedures of the study

In this in vivo experiment, either-sexed Wistar rats weighing 200-250g were involved. Regular standard diet feedings were given to the animals. The rats were housed in typical lab settings with a light-dark cycle. Six to ten animals were included in each experimental group. The care of the animals was provided in accordance with criteria created by the “New Delhi-based Indian National Science Academy (INSA), and the study procedure was approved by the Institutional Animal Ethic Committee (IAEC)”. Figure 1 depicts the experimental procedure of the study.

The chemicals ovalbumin, L-arginine (L-Arg), L-ascorbic acid (L-AA), N-nitro-L-arginine methyl ester (L-NAME), 7-nitroindazole (7-NI), and aminoguanidine (AG) were obtained from Sigma Aldrich Chemical Co., USA. All substances were purchased locally. The only exception was 7-NI, which was combined with a drop of Tween-80 and dissolved in double-distilled water. Freshly prepared drugs were administered intraperitoneally (i.p.) at a dose of 2 ml per kg of body weight.

Experimental procedure

On day 21, the treatments included a stress protocol, followed by vehicle treatments in the control group and ascorbic acid, L-arginine, 7-Ni, L-NAME, and AG in the research groups. On day 21 of the study period, blood samples were taken for various biochemical tests.

Stress

The experimental stressor was restraint stress (RSx1) for one hour on day 21. Rats were restrained in a customized Plexiglas Restrainer for restraint stress (INCO, Ambala, India).

Experiment methods

From various experimental groups, blood samples were taken. The Lowry method [23] was used to estimate protein, and the parameters listed below were measured:

Estimation of Malondialdehyde (MDA)

The technique was used to estimate MDA levels [24]. The reaction mixture consisted of 1.5 ml acetic acid (20%; pH 3.5), 0.1 ml plasma sample, 0.2 ml sodium lauryl sulfate (8.1%), and 0.8% thiobarbituric acid in an aqueous solution. 4 ml of distilled water was added, and the mixture was heated at 95°C for one hour. The mixture was centrifuged after the addition of 5 ml of n-butanol and pyridine (15:1 v/v) and 1 ml of distilled water. The results were expressed as μm/ml/mg protein using a spectrophotometer (UV 5740 SS, ECIL, India) to detect the absorbance of the organic layer at 532 nm.

Estimation of Reduced Glutathione (GSH) and Superoxide Dismutase (SOD)

The approach outlined by Ellman was used to estimate GSH levels [25]. Following the approach outlined by Nandi and Chatterjee [26], the levels of superoxide dismutase (SOD) were measured. The assay involved adding 0.02 ml of the sample, 0.02-0.08 ml of pyrogallol, and 0.1 ml of EDTA to 2.860 ml of tris buffer (50 mM) with a pH of 8.5 (30 mM). The mixture was then analyzed using a spectrophotometer to determine the increase in absorbance at 420 nm over a period of 30 seconds to 3 minutes. The initial 30 seconds served as a lag period for the pyrogallol reaction. To achieve an absorbance change rate of 0.025 to 0.030 per minute, the concentration of pyrogallol was adjusted. The addition of pyrogallol did not induce SOD to cause an increase in absorbance at 420 nm. 

Estimation of Catalase Levels

The activity of the enzyme catalase in plasma was assessed using the Aebi technique with hydrogen peroxide as the substrate [27]. A mixture was prepared consisting of 1 ml of hydrogen peroxide (30 mM/l), 1.9 ml of PBS at pH 7.0, and 0.1 ml of the plasma sample. The absorbance at 240 nm was monitored using a spectrophotometer (UV 5740 SS, ECIL, India) for a duration of 30 seconds. A decrease in absorbance was observed during this period. Catalase activity units were calculated per milligram of protein.

Estimation of Total Antioxidant Capacity (TAC)

As per Degraft-Johnson's method [28], the ferric reduction capacity of plasma (FRAP) was evaluated. A working solution consisting of 2.9 ml of FRAP reagent (acetate buffer: TPTZ: FeCl3) in a ratio of 10:1:1 was prepared. Further, 75 microliters of the plasma sample were added. Absorbance readings were taken at 593 nm, once at 0 minutes against a blank screen and again at 4-6 minutes. The results were expressed in units of M/L. 

Statistical analysis

Data analysis was performed using IBM Corp. Released 2013. IBM SPSS Statistics for Windows, Version 22.0. Armonk, NY: IBM Corp. The collected data were subjected to statistical analysis using Kruskal-Wallis, one-way ANOVA, and Tukey's tests. The results were reported as Mean ± SEM. To determine statistical significance, a probability level of 5% (p<0.05) was employed.

## Results

Effects of acute (RSx1) restraint stress on the concentrations of GSH and MDA in rats and their regulation by antioxidants and NO modulators

Significant alterations in oxidative markers were observed in the blood biochemical data of rats infected with OVA and subjected to acute restraint stress (RSx1). Comparisons between different groups revealed that RSx1 increased malondialdehyde (MDA) levels and hindered the decrease in glutathione (GSH) levels under acute stress conditions. Analysis of blood oxidative stress markers in response to RSx1 showed a 34% reduction in GSH levels and a 51% elevation in MDA levels.

**Figure 2:**
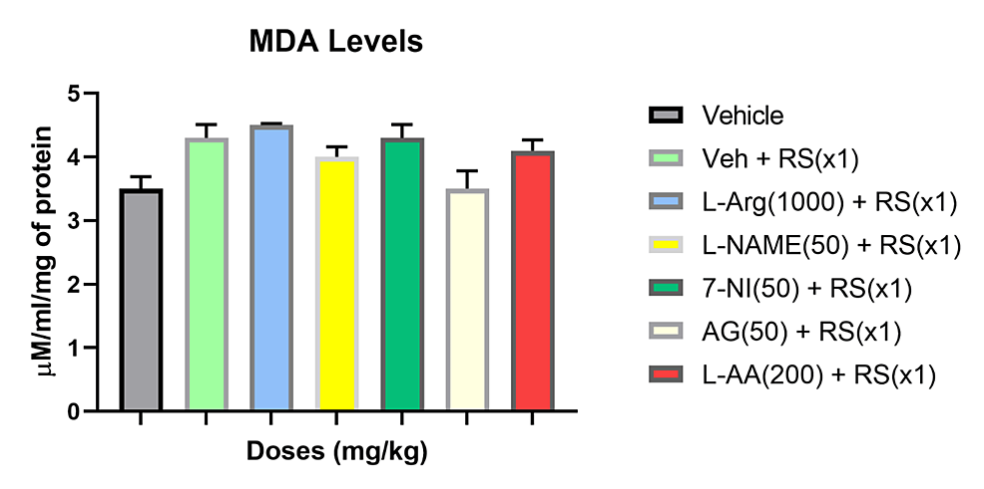
RSx1 impact and its modulation by NO modulators and antioxidants on MDA concentrations in rats

**Figure 3:**
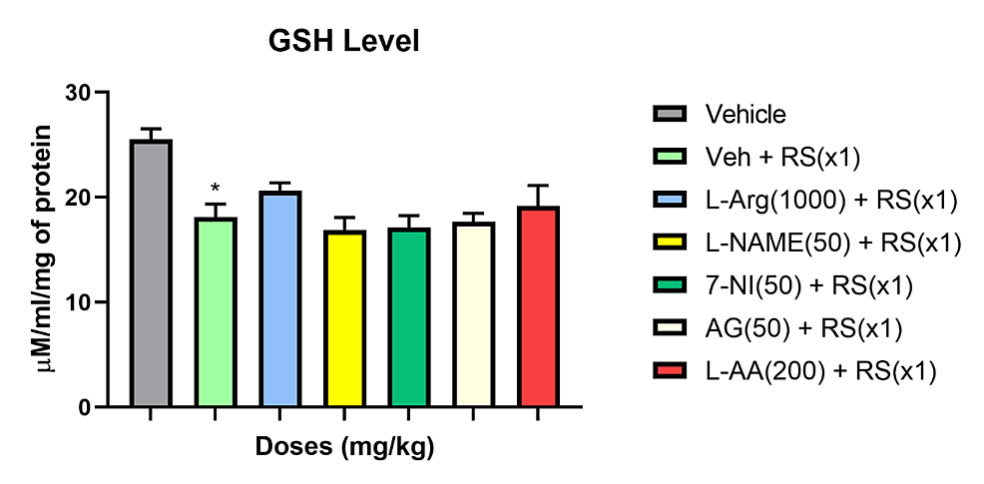
RSx1 impact and its modulation by NO modulators and antioxidants on GSH concentrations in rats * Significant (p ˂ 0.05), the change compared to vehicle-group for GSH Data are articulated as Mean ± SEM (n=8)

Treatment with the NO precursor L-Arginine (1000 mg/kg) was found to reverse the plasma levels of MDA and GSH induced by RSx1, contrasting the Vehicle + RS group. However, the levels of MDA remained unaffected by L-NAME (50 mg/kg) and 7-NI (50 mg/kg) compared to the Vehicle + RS group (P>0.05). Pretreatment with the antioxidants L-Ascorbic acid (L-AA) (200 mg/kg) and AG (50 mg/kg) reduced the MDA levels in the acute stress condition compared to the RS group with vehicle pretreatment (P>0.05). Additionally, NO synthase inhibitors and antioxidants improved plasma GSH levels during acute stress compared to the vehicle + RS groups (P>0.05). Figures 2, 3 depicts the impact of acute restraint stress (RSx1) on the concentrations of MDA and GSH in the rats' blood, as well as how NO modulators and antioxidants modify these effects.

Catalase, SOD, and total antioxidant concentrations in rats during acute (RSx1) restraint stress, and how antioxidants and NO modulators affect these concentrations

The biochemical data analysis revealed that acute restraint stress (RSx1) significantly inhibited the activity of superoxide dismutase (SOD), catalase, and total antioxidant capacity (F (6, 48) = 22.7, P<0.001 for SOD; F (6, 48) = 5.4, P<0.05 for catalase; F (6, 48) = 29.5, P<0.001 for total antioxidant capacity) compared to the vehicle group in terms of the stress-induced alterations of these oxidative markers.

**Figure 4:**
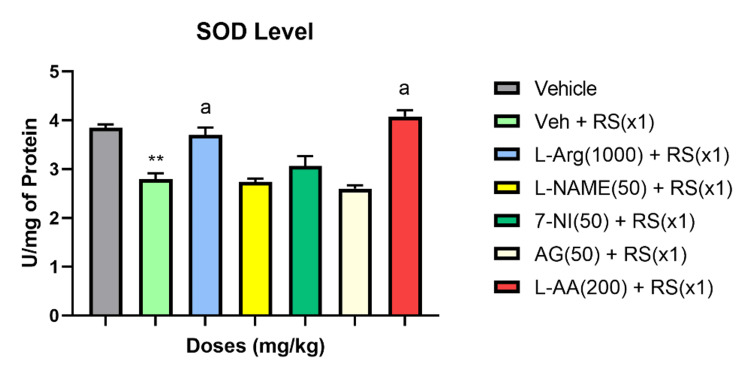
RSx1 impact and its modulation by antioxidant and NO modulators on SOD levels in rats ** Significant (p ˂ 0.01), the change compared to Vehicle-group for SOD a Significant (p ˂ 0.05), the change compared to the Vehicle + RS(x1)-group Data are articulated as Mean ± SEM (n=8)

**Figure 5:**
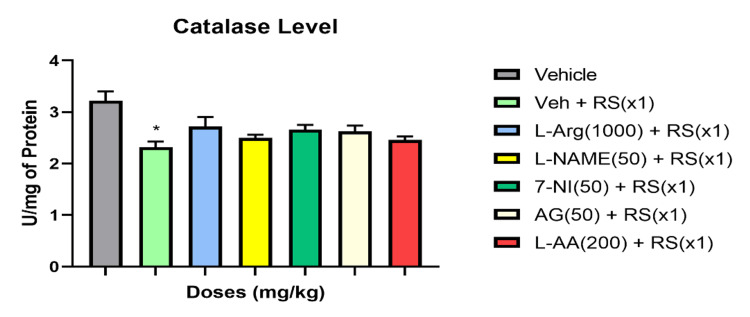
RSx1 impact and its modulation by antioxidant and NO modulators on catalase levels in rats * Significant (p ˂ 0.05), the change compared to vehicle-group for catalase Data are articulated as Mean ± SEM (n=8)

**Figure 6:**
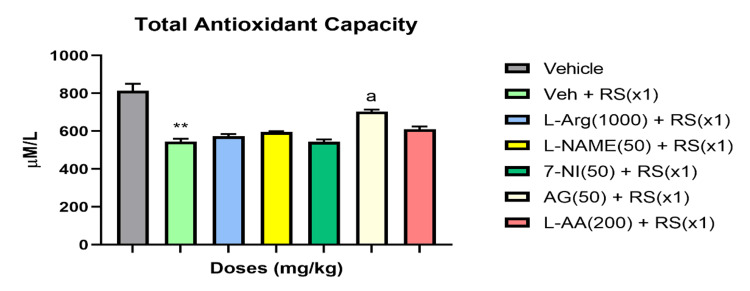
RSx1 impact and its modulation by antioxidant and NO modulators on Total Antioxidant Capacity levels in rats ** Significant (p ˂ 0.01), the change compared to Vehicle-group for SOD and Total Antioxidant Capacity a Significant (p ˂ 0.05), the change compared to Vehicle + RS(x1)-group Data are articulated as Mean ± SEM (n=8)

RSx1 led to a significant decrease in these oxidative stress indicators compared to the vehicle group (P<0.01 for SOD and total antioxidant capacity; P<0.05 for catalase). However, pretreatment with the NO precursor L-Arginine (1000 mg/kg) in the acute stress groups showed improvements in these oxidative parameters (P<0.01 for SOD; P>0.05 for catalase and total antioxidant capacity). Further, the NO-synthase inhibitors 7-NI (50 mg/kg), L-NAME (50 mg/kg), and AG (50 mg/kg) significantly suppressed the levels of total antioxidant capacity compared to the Vehicle + RS group (P<0.01), exacerbating the effects of stress on these oxidative stress parameters for catalase, SOD, and total antioxidant capacity levels. In contrast to the Vehicle + RS group, the antioxidant L-ascorbic acid (200 mg/kg) effectively restored SOD levels (P<0.01) and attenuated the RS-induced reductions in catalase and total antioxidant levels (P>0.05). Figures 4-6 illustrate the impact of acute restraint stress (RSx1) on the levels of catalase, SOD, and total antioxidants in the rat’s blood, as well as how antioxidants and NO modulators influence these levels.

## Discussion

Internal or external events that induce stress trigger the "activation of the sympathetic nervous system (SNS) and the hypothalamic-pituitary-adrenal (HPA) axis". These stress responses generate numerous signals that elicit a range of physiological and mechanistic alterations in the body. These adaptations aid in maintaining homeostasis through the body's regulatory processes [29].

Free radicals are very reactive molecules that are important in both health and disease. The two primary elements of the most intricately related mechanism for maintaining homeostasis are reactive oxygen species (ROS) and reactive nitrogen species (RNS). "NO modulators have been effectively employed as experimental tools to research NO-ergic pathways in both experimental and clinical situations" since nitric oxide (NO) is a special chemical with a diversity of effects [30,31]. Free radicals are signaling particles whose excessive synthesis damages biologically derived materials and causes several morbidities [32].

Cancer and diabetes mellitus are two conditions caused by "mitochondrial oxidative stress," which has been linked to pro-oxidants. It has long been known that oxidative stress plays a role in several pathological conditions that lead to cardiovascular disease, as well as other morbidities such as cancer, neurological disorders, diabetes, ischemia/reperfusion, and aging. "Inflammatory oxidative conditions" are a group of diseases that include chronic inflammation, atherosclerosis, and damage from ischemia and reperfusion [33-35]. An increase in oxidative damage to lipids, DNA, and protein pathways causes free radicals effects on aging. Free radicals have a lot of pathological involvement in a multi-biological ecosystem; that's why they are called powerful particles [36].

Reactive oxygen and nitrogen species both have important roles to play in these situations. The results of the studies showed how crucial oxidative stress is to a number of clinical morbidities. To gain control over these modalities, it may be appropriate to maintain the balance between these two routes, ROS and RNS [37]. Free radicals are thought to be extremely harmful in the emergence of many pathological illnesses, such as cancer, inflammation, and neurological disorders [38-40]. The endogenous antioxidant system feeds them electrons to stabilize them and halt further oxidation. The imbalance of pro- and antioxidant activity is a sophisticated instrument for multiple modulations. The substantial data showed how important ROS are in the control of several modifications that are neurobehavioral in nature and are mediated by stress. By giving free radicals electrons, antioxidant defense mechanisms limit the endogenous harm that they can cause. Several studies have reported that impacts on different pathological situations depend on the preservation of the equilibrium between the creation of pro-oxidant and antioxidant systems [41,42].

Through hormonal and neurotransmitter abnormalities as well as cellular damage brought on by free radicals and the intermediates of their interactions, stress is connected to an increase in oxidant formation and oxidative damage [14]. Depending on the kind, length, and severity of the stressor, oxidative damage may develop to varying degrees [15].

Antioxidants were investigated in research by Pal et al. to see how they impacted the effects of restraint stress (RS) on rats [43]. According to biochemical evidence, RS increased the levels of MDA in the blood and brain, whereas antioxidant therapy reduced these levels. Pharmacological and biochemical data suggest that free radicals may be to blame for these neurobehavioral effects of stress [43]. Reactive oxygen species may serve as a mediator for stress-induced immunomodulation, according to research by Pal R et al. As seen by decreased levels of (a) splenic plaque-forming cell counts, (b) anti-SRBC antibody titre, (c) IFN and IL-4, RS substantially suppressed both humoral and cell-mediated immune responses, and (d) footpad thickness response. Blood tests for oxidative stress markers showed that RS dramatically elevated plasma corticosterone levels as well as levels of malondialdehyde and reduced glutathione, the consequences of lipid peroxidation. As a result, it was also shown that the antioxidant enzymes catalase and superoxide dismutase were less active [44].

Kamper EF et al. conducted a study utilizing Wistar rats to investigate the effects of chronic moderate stress (CMS), a widely used animal model of depression, on markers of oxidative homeostasis-allostasis and sICAM-1, an indicator of endothelial damage. The experiment's findings showed that CMS had varied effects on the levels of oxidative stress and compensatory mechanisms in the two sexes, most likely because of variations in the systems that regulate oxidant/antioxidant pathways [45]. Chronic psychological stress increases tissue damage and oxidative stress in both humans and caged laboratory animals. Zafir A. et. al., investigated how stress hormones affected the oxidative processes in the brains of rodents. When people are under physical or mental pressure, stress hormones have been proven to actively induce oxidative stress in the brain. The study has significantly influenced the understanding of oxidative stress as a crucial pathological mechanism in chronic stress and has also provided valuable insights into the development of anti-stress therapeutic strategies [46].

Our biochemical studies to back up the altered immune function brought on by stress with modifications in oxidative stress markers suggest that acute stress increased MDA levels, altered GSH levels differently, reduced SOD and catalase levels, and decreased the capability of the body's antioxidant system. As seen by the much lower levels of GSH and significantly higher levels of MDA in comparison to the control group, acute stress greatly elevated the levels of lipid peroxidation. Glutathione has a protective role in immune system modulation. L-Arginine, a precursor to NO, successfully reversed these changes in MDA and GSH levels brought on by RS. L-NAME and 7-NI were found to have no effect on serum MDA levels under acute stress. MDA levels were decreased with the iNOS inhibitor AG and the antioxidant L-ascorbic acid. Antioxidants and NO-synthase inhibitors were observed to worsen the GSH levels in the acute stress groups.

The balance between levels of antioxidants and oxidants controls how a disease progresses. Oxidative stress could happen if this balance changes in favor of the oxidants. The presence of antioxidants is essential for counteracting the effects of free radicals. It is common knowledge that certain unpleasant stimuli make free radicals more active and regulate the onset of disease states. Two endogenous antioxidants and antioxidant enzymes, SOD and catalase, are crucial in controlling the production of free radicals. SOD, catalase, and total antioxidant levels decreased in the current study compared to the control group, indicating that acute stress generates an excessive quantity of free radicals (group without RS). SOD catalyzes and neutralizes the multiple metabolic pathways that result in superoxide radicals. An enzyme called catalase is responsible for catalyzing the oxidation of H2O2 into water and oxygen. Compared to the RS-treated vehicle group, L-arginine dramatically restored the RS-induced alterations in SOD, catalase, and total antioxidant capacity in the blood. This advantageous effect may have been mediated by nitric oxide's antioxidant mechanism. Different oxidative stress markers were negatively impacted by oxidative stress, which was increased by the NOS inhibitors L-NAME, 7-NI, and AG. The antioxidant L-ascorbic acid tends to increase SOD, catalase, and the overall antioxidant capacity of the blood during times of acute stress, in contrast to the vehicle group that received RS treatment. Free radicals are also scavenged by it. It's noteworthy to notice that antioxidants like alpha tocopherol and N-acetylcysteine have effects that are pretty comparable to the propensity to attenuate acute stress-induced changes that was observed in one of our lab's earlier studies [47]. This underscores even more how important ROS-RNS interactions are to this occurrence. Furthermore, glutathione levels, SOD, GST, and catalase activity, as well as lipid peroxidation, were found to be increased, SOD, GST activity was increased, and catalase activity was decreased by post-stress vitamin E therapy more than vitamins A and C.

Vitamin E supplementation is known to effectively counteract the production of free radicals in brain tissues and alleviate oxidative stress [22]. Another study demonstrated the potential of alpha-lipoic acid as a promising antioxidant option for treating lipid peroxidation induced by stress [48]. This underscores even more how important ROS-RNS interactions are to this occurrence. Furthermore, glutathione levels, SOD, GST, and catalase activity, as well as lipid peroxidation, were found to be increased, SOD, GST activity was increased, and catalase activity was decreased by post-stress vitamin E therapy more than vitamins A and C. By scavenging free radicals generated in the brain tissues, vitamin E can be used as a dietary supplement to lessen oxidative stress [22]. Further studies reported that alpha-lipoic acid was shown to be a superior alternative as an antioxidant for treating lipid peroxidation induced by stress [48].

Oxidative stress and free radicals are widely recognized as detrimental to human well-being [49]. A plethora of research indicates that free radicals indeed play a role in the onset and progression of various ailments, spanning from cardiovascular diseases to cancer. Antioxidants, a category of compounds capable of counteracting oxidative stress and ameliorating its impact on human health, have garnered significant attention within the biomedical research community [49]. These compounds have not only exhibited substantial efficacy in terms of preventing and/or treating diseases but have also been perceived as having minimal adverse effects. While antioxidants undoubtedly offer valuable contributions to averting, managing, or treating human pathologies, it is imperative to acknowledge that they are not entirely devoid of potential negative repercussions [49]. Conversely, certain prooxidant compounds or agents can also have benefits for human well-being, particularly in the realm of cancer therapy. In summary, oxidative stress, while undeniably a leading detriment to individual wellness and health, can also be harnessed as a therapeutic tool, provided that precise modulation of this process within the human body can be achieved.

## Conclusions

In conclusion, this study demonstrates the significant role of oxidative stress in the pathogenesis of various diseases and highlights the potential of antioxidant therapy as a viable treatment option. The results obtained from Wistar rats subjected to acute restraint stress (RSx1) reveal that oxidative stress markers, including malondialdehyde (MDA) and glutathione (GSH), were significantly altered. The levels of MDA, an indicator of lipid peroxidation and oxidative damage, were increased, while GSH, a key antioxidant molecule, was decreased under acute stress conditions. However, the administration of antioxidants such as L-ascorbic acid (L-AA) and nitric oxide (NO) modulators, including the NO precursor L-arginine, demonstrated promising effects in mitigating the oxidative stress induced by acute restraint stress. L-AA and L-arginine were able to restore the levels of SOD, catalase, total antioxidant capacity, MDA, and GSH, indicating their potential as therapeutic agents in reducing oxidative stress. Further, the NO synthase inhibitors (L-NAME, 7-NI, and AG) showed mixed results, with some parameters being improved and others exacerbated. These findings suggest the complex role of NO in modulating oxidative stress and the need for further investigation to elucidate its precise mechanisms.

Targeting and reducing oxidative stress through antioxidant therapy and NO modulation holds potential for the treatment of oxidative stress-related diseases. Further research is warranted to better understand the underlying mechanisms and optimize the use of antioxidants and NO modulators in clinical settings.
